# Early angiographic changes after hemostatic radiotherapy for gastric cancer bleeding, mentioning the mechanism and potential immediate effects of the treatment

**DOI:** 10.1007/s10120-025-01616-8

**Published:** 2025-05-02

**Authors:** Yuki Wada, Motoko Konno, Tomoki Tozawa, Tomochika Sato, Tetsugaku Shinozaki, Satoshi Kumagai, Noriko Takagi, Koji Fukuda, Hiroyuki Shibata, Naoko Mori

**Affiliations:** 1 Department of Radiology, Akita University Graduate School of Medicine, 1-1-1 Hondo, Akita, 010-8543 Japan; 2 Department of Clinical Oncology, Akita University Graduate School of Medicine, 1-1-1 Hondo, Akita, 010-8543 Japan

**Keywords:** Stomach neoplasm, Gastrointestinal hemorrhage, Radiotherapy, Minimally invasive procedure

## Abstract

Although hemostatic radiotherapy has been reported as an effective treatment for gastric cancer bleeding, its mechanism and immediate effects remain unclear. We experienced a case of gastric cancer bleeding originating from both the whole gastric tumor and a left gastric arterial pseudoaneurysm at the tumor-associated ulcer. The patient was treated with radiotherapy for bleeding from the whole gastric tumor, followed by transcatheter arterial embolization for the left gastric arterial pseudoaneurysm. Angiography performed two hours after radiotherapy with an X-ray of 8 Gy in a single fraction revealed the disappearance of both tumor vessels and tumor stain from not only the embolized left gastric artery but also both the non-embolized right gastric artery and common trunk of the left gastric and the left hepatic arteries, which indicated these angiographic changes of the non-embolized arteries were presumed to reflect an immediate effect of hemostatic radiotherapy. Following hemostatic treatments, the patient’s vital signs stabilized, and hemoglobin levels did not decrease, indicating immediate hemostasis. This case suggests a link between hemostatic mechanism and early tumor vessel changes, indicating that hemostatic radiotherapy can achieve rapid bleeding control. Therefore, hemostatic radiotherapy should be considered an emergency treatment option for gastric cancer bleeding.

## Introduction

Approximately 60% of patients with non-resectable gastric cancer require intervention due to tumor-related complications [1]. Among these, tumor bleeding is one of the most severe, often necessitating blood transfusion, hospitalization, and sometimes resulting in death [2]. Hemostatic local therapy for gastric cancer bleeding includes surgery, endoscopic hemostasis, transcatheter arterial embolization (TAE), and hemostatic radiotherapy. Hemostatic radiotherapy has been reported to achieve a high hemostatic rate of 80–90% with minimal severe adverse events [3–5]. However, its mechanism and immediate effects remain unclear.

We experienced a case with gastric cancer bleeding from both the whole tumor and a left gastric arterial pseudoaneurysm located at the tumor-associated ulcer. The patient underwent hemostatic radiotherapy to control bleeding from the whole gastric tumor, followed by TAE for the pseudoaneurysm. This sequential approach allowed for the evaluation of early angiographic changes immediately after hemostatic radiotherapy. Angiography performed two hours after radiotherapy revealed the disappearance of both tumor vessels and tumor stain from not only the embolized left gastric artery but also non-embolized arteries, which was presumed to reflect an immediate effect of hemostatic radiotherapy. Based on laboratory data and physical findings observed after hemostatic radiotherapy and subsequent TAE, the immediate hemostatic effect of radiotherapy was thought to be achieved within a few hours. This case provides insights into the mechanism and rapid effectiveness of hemostatic radiotherapy. Herein, we present the case along with a literature review.

## Case presentation

The patient is a 57-year-old male with advanced gastric cancer. Two years ago, he experienced difficulty swallowing and was subsequently diagnosed with gastric cancer, classified histologically as moderately differentiated tubular adenocarcinoma. Due to the presence of peritoneal dissemination, gastrectomy was not considered; instead, systemic chemotherapy was administered at another hospital. Neither over-expression of HER2 nor *HER2* gene amplification was observed. Microsatellite instability was not detected. Comprehensive genomic profiling using FoundationOne^®^ showed *MTAP* loss and *KRAS* amplification; copy number of *KRAS* was seven. Chemotherapy with Oxaliplatin + Tegafur/Gimeracil/Oteracil, Nivolumab + Oxaliplatin + Tegafur/Gimeracil/Oteracil, Ramucirumab + Irinotecan, and then Nab-paclitaxel were conducted sequentially. Finally, LUNA18, a cyclic peptide inhibiting both mutant and wild type forms of *RAS* was administered as a phase 1 clinical trial. Two weeks before experiencing gastric cancer-related bleeding, a routine computed tomography (CT) scan revealed new metastases in the iliac bone and the liver; hence, his gastric cancer was diagnosed as a progressive disease. The clinical trial regimen was terminated, and further treatment options were under evaluation at the time.

**Fig. 1:**
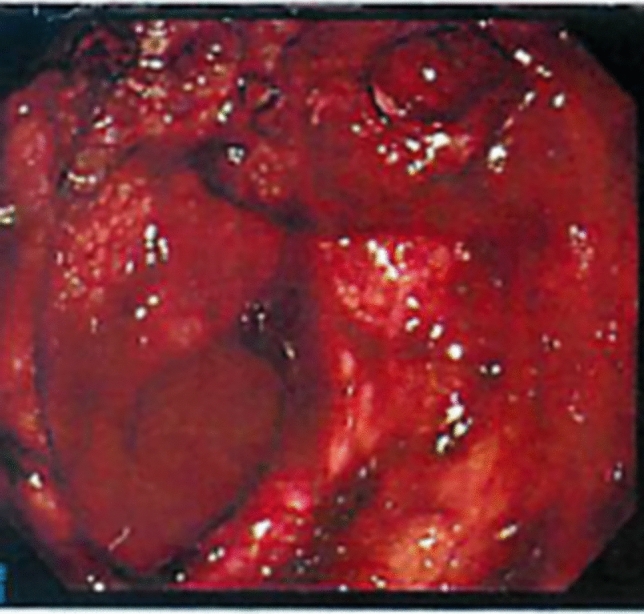
The upper gastrointestinal endoscopy on the day of bleeding from gastric cancer showed both exudative and pulsatile bleeding from the tumor at the lesser curvature of the stomach. Hemostatic points could not be identified, and endoscopic hemostasis was not feasible

**Fig. 2:**
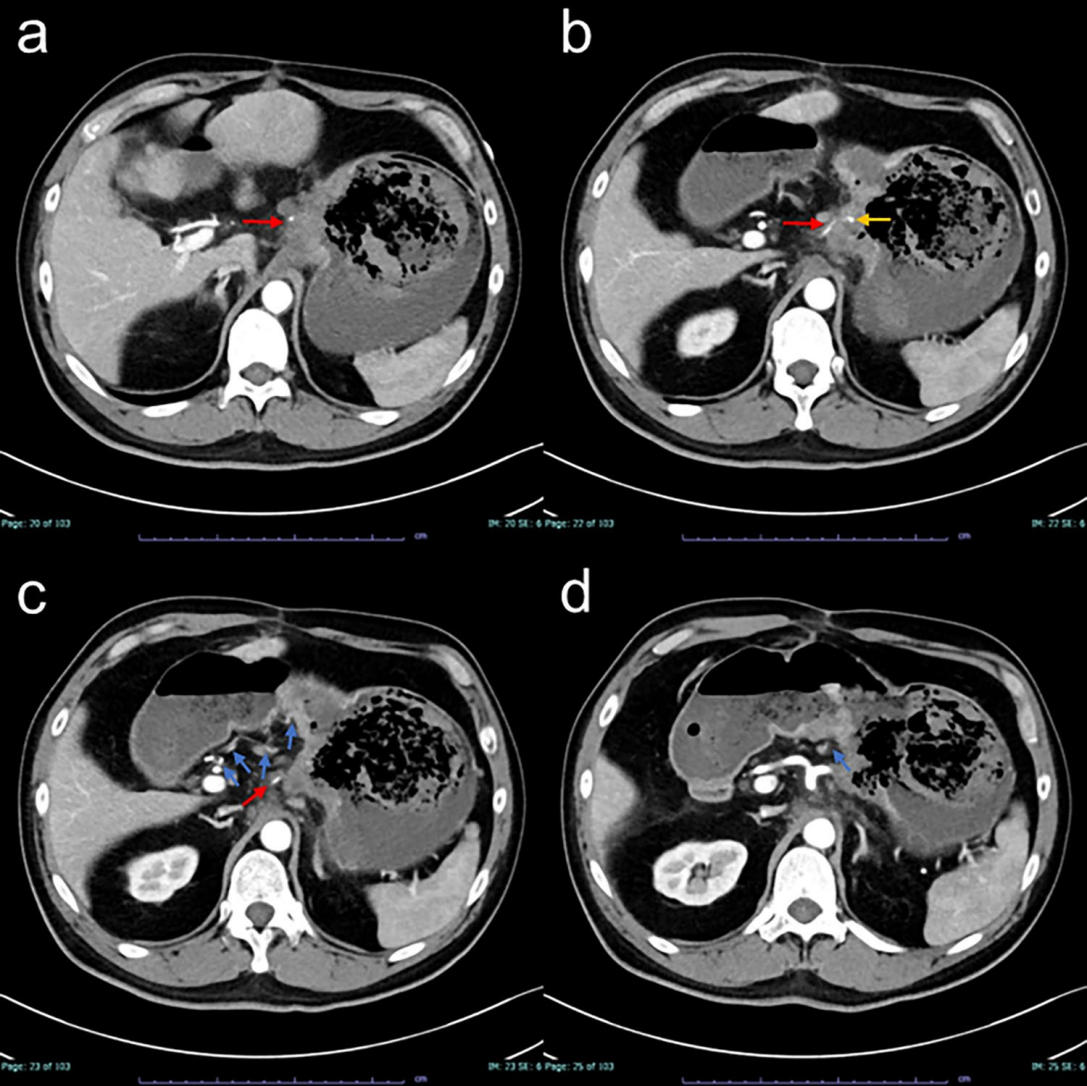
The arterial phase contrast-enhanced computed tomography images on the day of bleeding from gastric cancer (**a**–**d**, from cranial to caudal side). The arrows indicate the common trunk of the left gastric and the left hepatic arteries in red, the right gastric artery in blue, and the pseudoaneurysm in yellow. The pseudoaneurysm originated from the left gastric artery. The gastric tumor located at the lesser curvature of the stomach was blood supplied by both the left and the right gastric arteries

On the day of the gastric cancer-related bleeding, the patient presented to the hospital with symptoms of severe anemia. Laboratory data revealed a notable drop in hemoglobin levels from 9.4 mg/dL on the previous day’s routine test to 6.0 mg/dL. An emergency upper gastrointestinal endoscopy (Fig. 1) and contrast-enhanced CT imaging (Fig. 2) identified both exudative bleeding from the whole tumor at the lesser curvature of the stomach and pulsatile bleeding from a pseudoaneurysm of the left gastric artery at the tumor ulcer. The tumor blood was supplied by both the right gastric artery and the common trunk of the left gastric and the left hepatic arteries. Due to the hematoma in the stomach, the exact hemostatic points could not be identified by gastrointestinal endoscopy, making endoscopic hemostasis unfeasible. Following a blood transfusion, the patient was transferred to our hospital for multidisciplinary hemostatic treatment. On arrival, blood pressure was 120/60 mmHg, the heart rate was 103 beats per minute, and SpO_2_ was 100% with 6 L per minute of oxygen, and the state of consciousness was clear. Although the pseudoaneurysm required TAE, this treatment is only effective for arterial bleeding and could not address the diffuse exudative hemorrhage from the tumor. Furthermore, approximately one hour was needed to prepare for TAE. Given these circumstances, we decided on an emergency hemostatic radiotherapy session to control the exudative bleeding from the tumor, followed by TAE for pulsatile bleeding from the pseudoaneurysm of the left gastric artery.

**Fig. 3:**
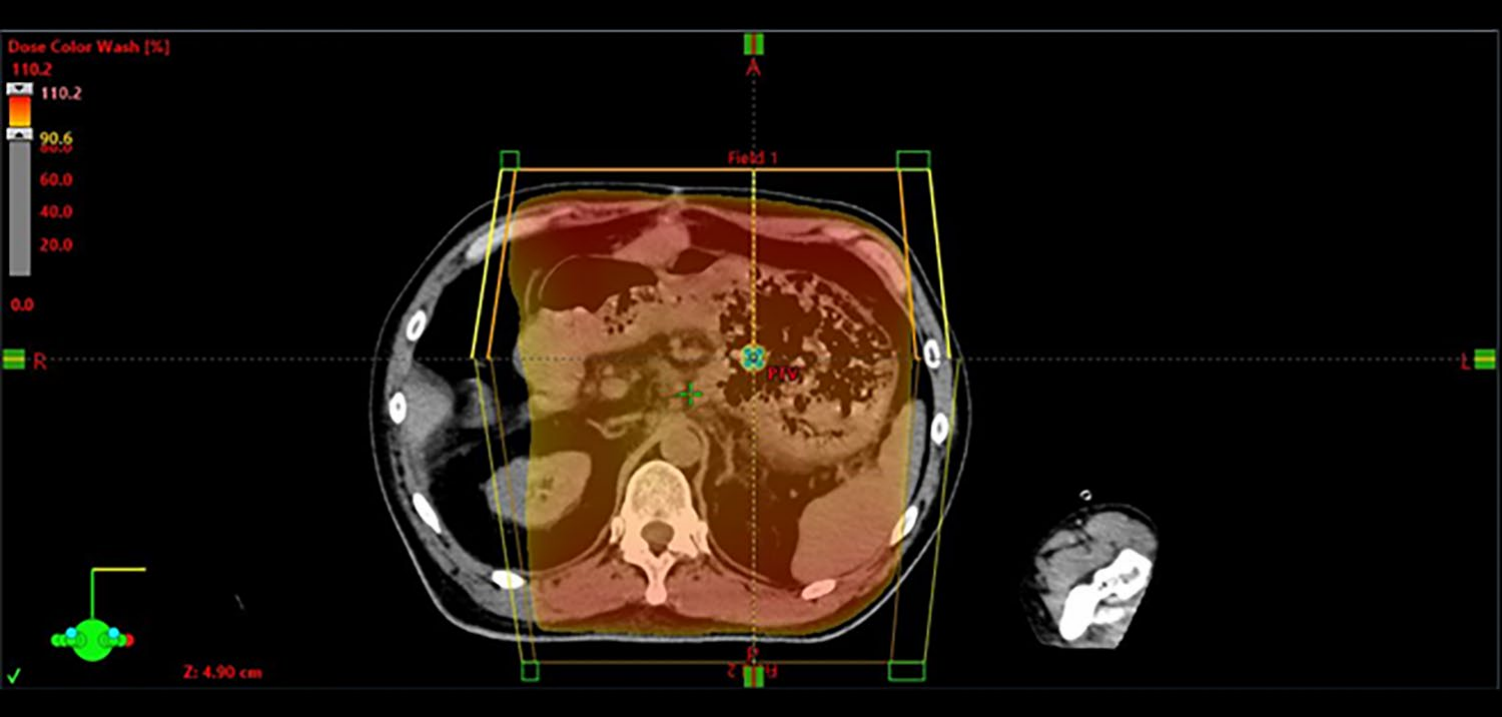
Key images of the hemostatic radiotherapy planning. Two opposed radiation fields from the anteroposterior direction were used, delivering 10 megavoltage X-rays of 8 Gy in a single fraction to the whole stomach

Emergency hemostatic radiotherapy was initiated 30 min after the arrival of the patient at our hospital. The treatment was delivered using two opposed irradiation fields from the anteroposterior direction, administering a single fraction of 8 Gy with 10 megavoltage X-rays to the entire stomach (Fig. 3).

**Fig. 4:**
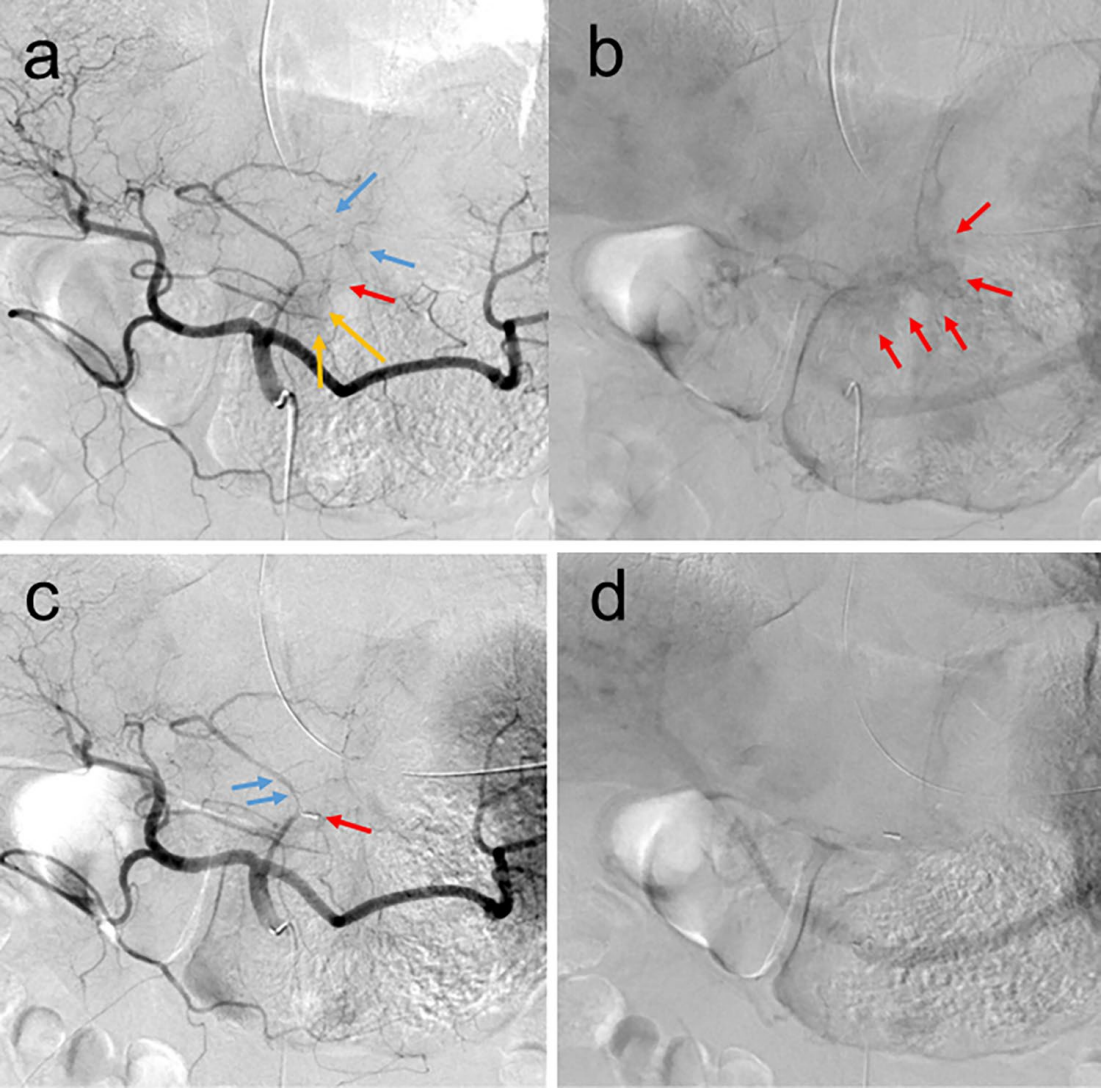
Angiographic images of transcatheter arterial embolization (TAE) were performed shortly after hemostatic radiotherapy. Arterial (**a**) and late phase (**b**) of the celiac trunk angiography, 1 h after hemostatic radiotherapy, revealed tumor vessels from the left gastric artery (**a**, red arrow), the right gastric artery (**a**, yellow arrows), and the common trunk of the left gastric and the left hepatic arteries (**a**, blue arrows). Tumor stain was also observed (**b**, red arrows). Arterial (**c**) and late phase (**d**) of the celiac trunk angiography, 2 h after hemostatic radiotherapy when finishing TAE of the pseudoaneurysm of the left gastric artery, revealed the selective embolization of the left gastric artery (**c**, red arrow), conserved blood flow of the common trunk of the left gastric and the left hepatic arteries (**c**, blue arrows), and the disappearance of both tumor vessels and tumor stain from the right gastric artery and the common trunk

Immediately after the radiotherapy session, the patient was transferred to the angiography room. Celiac trunk angiography before embolization, which was performed one hour later from the hemostatic radiotherapy, revealed the presence of tumor vessels and a tumor stain from both the left and right gastric arteries (Fig. 4a, b). The left gastric artery and the left hepatic artery formed a common trunk, which also supplied blood to the tumor (Fig. 4a, blue arrows). The pseudoaneurysm of the left gastric artery was embolized using micro-coils selectively, and the common trunk and the left hepatic artery could be conserved. A follow-up celiac trunk angiography after embolization, which was performed two hours after hemostatic radiotherapy, revealed the disappearance of both tumor vessels and the tumor stain from the right gastric artery and the common trunk (Fig. 4c, d). Given that no embolization was performed for the right gastric artery and the common trunk, the disappearance of the tumor vessels and the tumor stain was presumed to reflect an immediate effect of hemostatic radiotherapy.

**Fig. 5:**
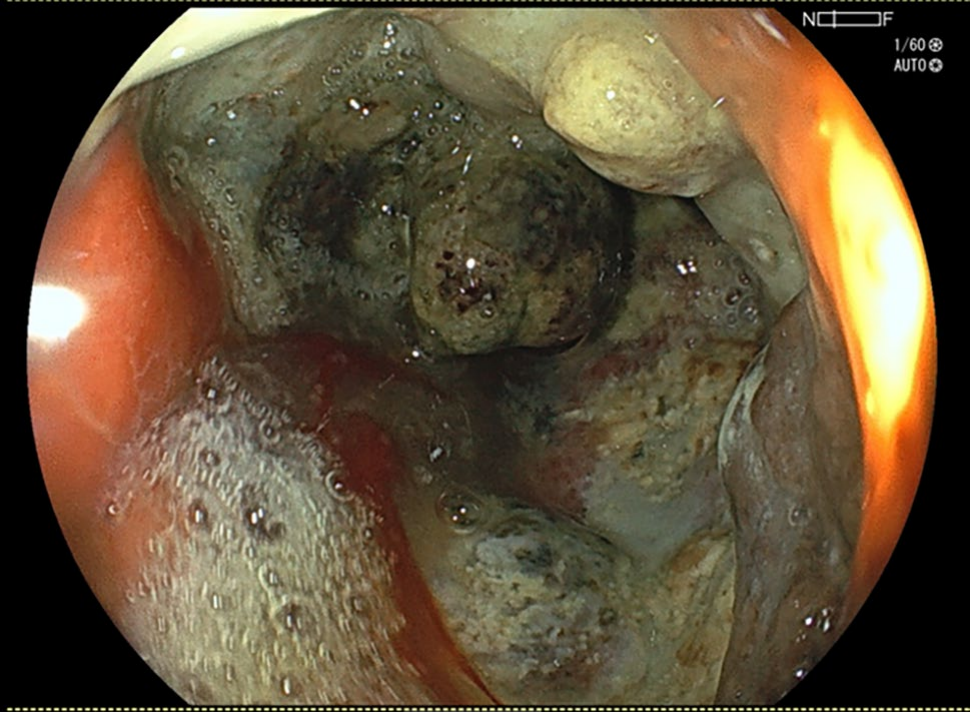
The upper gastrointestinal endoscopy 10 days after hemostatic treatments showed favorable bleeding control from gastric cancer

After hemostatic radiotherapy followed by TAE, the patient’s vital signs stabilized, and periodic blood tests showed no further decline in hemoglobin levels. This suggested that hemostatic radiotherapy had achieved an immediate hemostatic effect within a few hours. A follow-up upper gastrointestinal endoscopy 10 days after the hemostatic therapies revealed no active bleeding from the gastric cancer (Fig. 5). With improvement in his overall condition, the patient was transferred back to the previous hospital for systemic chemotherapy.

## Discussion

In the present patient, the disappearance of both tumor vessels and a tumor stain from the right gastric artery and the common trunk of the left gastric and the left hepatic arteries, for which no embolization was performed, was observed within a few hours after hemostatic radiotherapy. Furthermore, physical findings and laboratory data indicated an immediate hemostatic effect. These findings suggest that hemostatic radiotherapy may have contributed to the rapid disappearance of tumor vessels.

The hemostatic mechanism of radiotherapy for gastric cancer bleeding remains not clearly understood. Previous studies have hypothesized several potential mechanisms, including changes in tumor vessels, radiation-induced platelet aggregation, tumor shrinkage, and fibrosis of the tumor and surrounding tissues [6–9]. However, to the best of our knowledge, no reports have clearly demonstrated the mechanism of hemostatic radiotherapy in vivo. Conventional radiotherapy with 1.8–3.0 Gy per fraction causes genetic damage to irradiated cells, leading to tumor shrinkage, and fibrosis of the tumor and surrounding tissues for a few days to months. In contrast, hypofractionated radiotherapy with a high dose per fraction can directly cause vascular changes in tumor vessels by damaging vascular endothelial cells within a few hours to days, mildly at 5–10 Gy per fraction and severely at 10 Gy or more per fraction [10, 11]. Therefore, a higher radiation dose per fraction can cause a significantly stronger and earlier impact on tumor vessels directly. However, a radiation dose per fraction cannot be increased without an upper limit because a higher radiation dose per fraction also causes more severe damage to normal tissues and organs. The frequently used radiotherapy regimens in a multicenter prospective study of hemostatic radiotherapy for gastric cancer conducted in Japan were 8 Gy in a single fraction, 20 Gy in 5 fractions (4 Gy per fraction), and 30 Gy in 10 fractions (3 Gy per fraction) [5]. Therefore, 8 Gy in a single fraction, which is also used in the present patient, is considered a safe upper dose at present when hemostatic radiotherapy for gastric cancer is performed with a higher dose in a single fraction. The angiographic images obtained in the present patient may indicate that the disappearance of tumor vessels and the tumor stain occurred within a few hours after radiotherapy, suggesting a possible association with the hemostatic effect of radiotherapy.

The immediate effects of hemostatic radiotherapy for gastric cancer bleeding have not been extensively researched. In the case of painful bone metastases, where palliative radiotherapy is often employed, the median time from radiotherapy to the onset of pain relief is reported to be 2–4 weeks [12, 13]. Therefore, many clinicians may assume that hemostatic radiotherapy also requires a similar timeframe to take effect. To date, two prospective studies have investigated hemostatic radiotherapy for gastric cancer bleeding, with initial evaluations conducted 2–4 weeks after treatment [3, 5]. Consequently, no prospective data is available on its hemostatic effects in the early phase after radiotherapy. However, a retrospective analysis showed that hemostasis was achieved at a median of 2 days, with some patients experiencing hemostatic effects as early as the following day after radiotherapy [14]. In this present case, hemostasis was achieved on the same day of radiotherapy, and this fact was consistent with the observed changes of tumor vessels and tumor stain in the angiography after hemostatic radiotherapy. This suggests that bleeding control can be achieved immediately in some patients following radiotherapy. Therefore, emergency radiotherapy should be considered a life-saving option for patients with severe gastric cancer bleeding when other local hemostatic treatments are not feasible.

In conclusion, we reported a case of gastric cancer bleeding in which both tumor vessels and the tumor stain disappeared, and an immediate hemostatic effect was achieved shortly after hemostatic radiotherapy followed by TAE. Our findings suggest that the hemostatic mechanism of radiotherapy is related to early changes of tumor vessels and tumor stains. Given that some patients, including those in our case and previous studies, can achieve immediate hemostasis, hemostatic radiotherapy should be considered a viable emergency treatment option for gastric cancer bleeding.
